# Governance of Tunisian sports organizations: what is the matter?

**DOI:** 10.3389/fspor.2025.1556256

**Published:** 2025-07-17

**Authors:** Samir Ghodhbani, Nizar Souissi

**Affiliations:** ^1^ Physical Activity, Sport and Health, University of Sfax, Sfax, Tunisia; ^2^ Department of Physical Education, Higher Institute of Sport and Physical Education of Ksar Said, University of La Manouba, Tunis, Tunisia; ^3^ Physical Activities, Sports, and Health Research Unit, National Sports Observatory, Tunis, Tunisia

**Keywords:** governance, nonprofit organizations, organizational performance, sports federations, stakeholder engagement, strategic leadership, governance models, accountability

## Abstract

**Introduction:**

Tunisian Sports federations with a public service mission are tasked with organizing, promoting, and developing their respective sports disciplines. Over the last decade, these federations, like other nonprofit organizations, have faced profound challenges regarding their governance methods. This study aims to first explore and categorize the different modes of governance within sports federations and, second, to examine how these governance models impact organizational performance.

**Design/Methodology/Approach:**

To identify and analyze the various governance modes within non-profit sports organizations, **adopting a positivist epistemological stance** and a **hypothetico-deductive approach**, this research was conducted on 20 national sports federations, utilizing a **questionnaire** directed at the president, general secretary, national technical director, and members of the executive committee of each federation. In addition, to analyze the data, an **exploratory factor analysis** was carried out, allowing for a comprehensive dimensionality check of the variables to test the hypotheses.

**Results:**

The findings indicate that in 33% of federations, strategic decisions and managerial operations are made through coordination between the various actors. In 24% of federations, a **couple** or **exploded mode** of presidential governance prevails. In the remaining federations, the president assumes the role of the sole decision-maker. Paradoxically, the results suggest that regardless of the governance mode in place, all four models positively influence the organizational performance of the federations.

**Discussion:**

This study demonstrates that all four governance models positively impact organizational performance in Tunisian sports federations. However, the strength and nature of these effects vary. The “managerial presidential” and “couple’s presidential” models show relatively stronger associations with performance outcomes, particularly in federations with established professional staff structures. These differences suggest that while each model contributes, their practical effectiveness depends on how clearly responsibilities are distributed and how decision-making is shared among key actors. This paper contributes critical insights into the relationship between governance models and organizational performance in the context of nonprofit sports organizations. The findings suggest that the implementation of an appropriate governance model can lead to significant improvements in both sports results and financial resources. National sports federations, especially members of the executive boards, stand to benefit from adopting governance models that align with their operational needs and strategic goals.

## Introduction

The concept of governance has traditionally been explored within the context of for-profit companies. However, the proliferation of financial scandals involving nonprofit organizations has shifted academic attention toward an emerging and critical research area: the governance of nonprofit associations (1), who proposed a governance-structure approach tailored to nonprofit organizations, particularly emphasizing the balance between volunteerism and professionalism.

This shift reflects the growing recognition of the importance of governance in ensuring transparency, accountability, and organizational sustainability in sectors beyond corporate entities. Since this pivotal transition, numerous scholars [e.g., (2, 3)] have delved into the complexities of governance within nonprofit organizations, recognizing its unique challenges and implications. Kreutzer (4) highlights that governance shapes how nonprofit organizations are directed, administered, and controlled, ultimately influencing their overall effectiveness and societal impact. Alm and Gammelsæter (5) provide a comparative framework for assessing governance standards, which can be used to benchmark Tunisian federations. Corporate social responsibility practices, as discussed by Anagnostopoulos and Shilbury (6), are relevant to nonprofit sport organizations seeking stakeholder legitimacy. Aguilera and Jackson (7) emphasize the cross-national differences in governance systems, which is pertinent when comparing Tunisian federations to international models.

**Figure 1:**
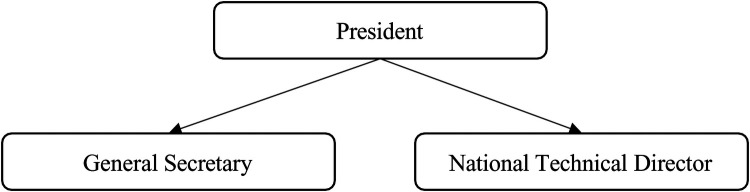
The strong presidential model.

**Figure 2:**
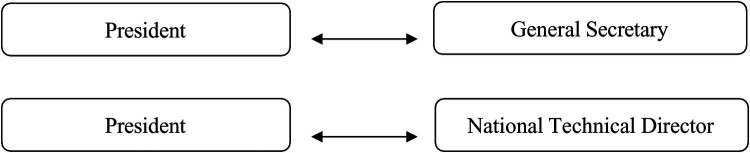
The “couple’s presidential” model.

**Figure 3:**
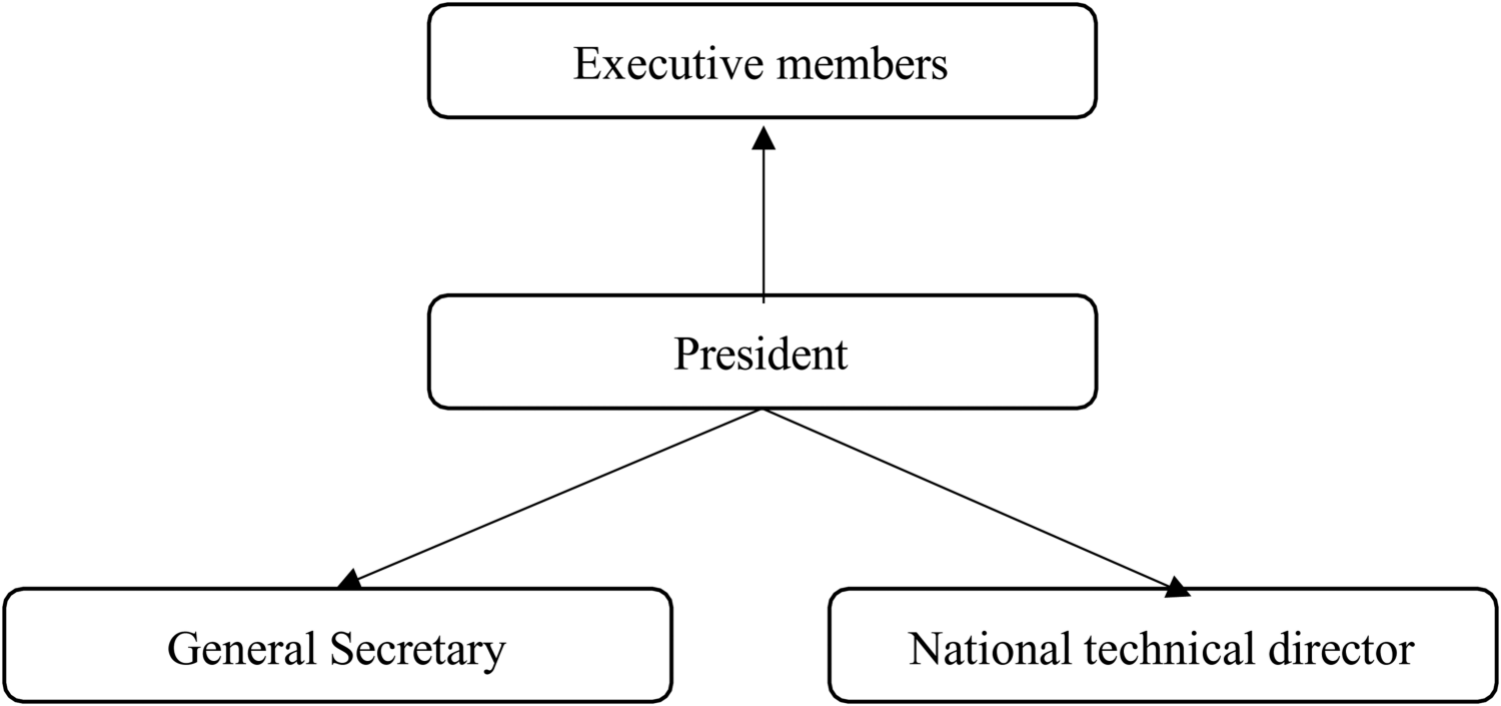
The “exploded presidential” model.

**Figure 4:**
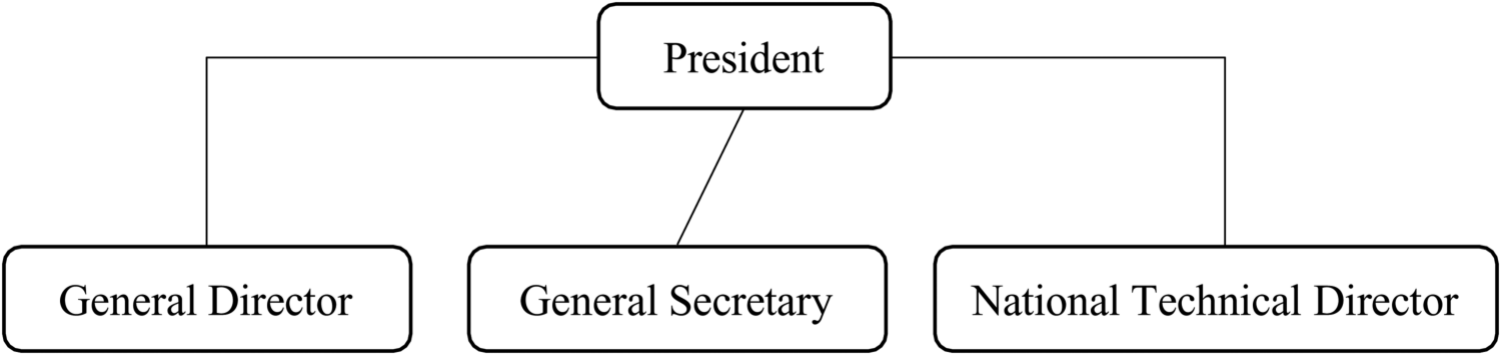
The managerial presidential model.

**Table 1:** Distribution of respondents by gender, age and experience.

Gender		Experience			Age		
Women	Men	Under 05 years	Between 5 and 10 years	Plus 10 years	Under 30 years old	Between 30 and 40 years old	Plus 40 years old
10	70	31	44	5	51	15	14

**Table 2:** Distribution of sports federations by model of governance.

Model of governance	Sports fédération
• Strong presidential	boxing, football, judo, fencing, gymnastic
• Couple's presidential	baseball, tennis, table tennis, handball, golf, rowing
• Exploded presidential	jujitsu, weightlifting, wrestling, karate, regby
• Managerial presidential	basketball, handball, taekwondo, volleybal

**Table 3:** Reliability test of the measurement scale of each model of governance.

Model of governance	Reliability statistics	
	Cronbach's alpha	*N* of items
The strong presidential model	0.786	3
The managerial presidential model	0.973	3
The exploded presidential model	0.909	3
The couple's presidential model	0.901	3

**Table 4:** Regression test for each model of governance.

Model summary				
Model	R	R square	Adjusted R square	Std. error of the estimate
1	.525^a^	0.401	0.112	0.98606693
^a^Predictors: (Constant), “strong presidential” model				
1	.660^a^	0.426	0.113	0.9934541
^a^Predictors: (Constant), “couple’s presidential” model				
1	.520^a^	0.449	0.136	0.98165229
^a^Predictors: (Constant), “exploded presidential” model				
1	.657^a^	0.403	0.11	0.70475957
^a^Predictors: (Constant), “managerial presidential” model				

**Table:** 

ANOVA^b^						
Model		Sum of squares	Df	Mean square	F	Sig.
1	Regression	2.051	1	2.051	3.05	.004^a^
	Residual	76.949	78	1.012		
	Total	79	79			
^a^Predictors: (Constant), “strong presidential” model						
^b^Dependent Variable: Organisational performance						
1	Regression	2.018	1	2.018	2.044	.000^a^
	Residual	76.982	78	0.987		
	Total	79	79			
^a^Predictors: (Constant), “couple’s presidential” model						
^b^Dependent Variable: Organisational performance						
1	Regression	3.836	1	3.836	3.981	.000^a^
	Residual	75.164	78	0.964		
	Total	79	79			
^a^Predictors: (Constant), “exploded presidential” model						
^b^Dependent Variable: Organisational performance						
1	Regression	4.256	1	4.256	3.253	.001^a^
	Residual	74.744	78	1.01		
	Total	79	79			
^a^Predictors: (Constant), “managerial presidential” model						
^b^Dependent Variable: Organisational performance						

**Table:** 

Model		Unstandardized coefficients		Standardized coefficients	*t*	Sig.
		B	Std. error	Beta		
1	(Constant)	3.99 × 10^−17^	0.112		0	1
	“strong presidential” model	0.525	0.113	0.525	2.224	0.004
^a^Dependent Variable: Organisational performance						
1	(Constant)	3.15 × 10^−17^	0.111		0	1
	“couple’s presidential” model	0.66	0.112	0.66	2.43	0
^a^Dependent Variable: Organisational performance						
1	(Constant)	3.61 × 10^−17^	0.11		0	1
	“exploded presidential” model	0.52	0.11	0.52	4.995	0
^a^Dependent Variable: Organisational performance						
1	(Constant)	3.96 × 10^−17^	0.112		0	1
	“managerial presidential” model	0.657	0.113	0.657	2.503	0.001
^a^Dependent Variable: Organisational performance						

The significance of governance within nonprofit sport organizations became evident in 1998 when a major scandal erupted in the sporting world. Members of the International Olympic Committee (IOC) were accused of accepting bribes from the Salt Lake City Organizing Committee to secure the 2002 Winter Olympic Games, following the city's unsuccessful bid for the 1998 Winter Games, awarded instead to Nagano, Japan. This scandal led to the expulsion of several IOC members and the implementation of new governance rules designed to enhance transparency and accountability. Often described as the “beginning of governance” in sport organizations, this case exemplifies how negative events can serve as catalysts for structural and cultural change.

The stakes in governance are especially high in sports due to the significant financial investments associated with major events like the Olympic Games, the FIFA World Cup, and the Tour de France. These events require robust governance frameworks to ensure integrity, mitigate risks, and prevent corruption. Unfortunately, subsequent scandals, such as the 2013 Lance Armstrong doping case—where systemic cheating was allegedly protected by the International Cycling Union (UCI)—and the 2015 “Fifagate” scandal involving money laundering, fraud, and corruption in the allocation of World Cup hosting rights, underscore persistent governance failures in the sector. Such practices not only tarnish the reputation of sports but also raise critical questions about accountability, ethics, and organizational sustainability.

While global scandals such as the IOC bribery case, the Lance Armstrong doping affair, and the 2015 “FIFA gate” corruption case occurred outside Tunisia, they have influenced perceptions and reforms locally. These international failures served as cautionary tales that underscored the need for greater transparency, ethical standards, and regulatory oversight within Tunisian federations. Consequently, Tunisia's recent governance reforms aim to avoid similar dysfunctions by reinforcing institutional integrity and public trust.

Governance failures have broader implications beyond reputational damage. Nonprofit sport organizations are heavily reliant on public trust and funding, with their financial support often coming from taxpayers, fans, and government authorities. As Winand (8) emphasizes, inadequate governance can jeopardize the survival of these organizations by undermining their legitimacy and risking the withdrawal of critical public funding. Consequently, ensuring strong governance is not only a matter of organizational integrity but also essential for securing long- term financial and social support.

The credibility of sport and its capacity to fulfill its broader societal roles depend significantly on the quality of governance. As the role of sport in society continues to expand—contributing to public health through physical activity, fostering social cohesion, and promoting educational and cultural values—the expectations of stakeholders regarding transparency and ethical conduct in nonprofit sport organizations are rising. Sport has the unique ability to transcend cultural and national boundaries, instilling fundamental values such as the rule of law, respect for others, freedom of expression, team spirit, solidarity, and fair play. However, these positive influences are compromised when governance lapses occur, eroding public trust and undermining the societal contributions of sport.

Sport carries a powerful normative dimension, promoting values such as fair play, inclusion, and respect for rules. These ideals are central to the “sport-for-development” literature, which positions sport as a tool for social cohesion and public good. Governance structures must reflect these values by ensuring transparency, participation, and accountability. Without this alignment, the credibility and broader societal role of sport organizations are at risk.

Given the growing complexity of the sector, it is essential to examine the models of governance within nonprofit sport organizations and their impact on organizational performance. This study seeks to explore the interplay between governance practices and performance outcomes, with a particular focus on identifying how different governance models influence the effectiveness, efficiency, and sustainability of nonprofit sport organizations. By addressing these questions, this research aims to contribute to the development of governance frameworks that enhance the credibility, functionality, and societal impact of sport organizations worldwide.

## About governance

Governance is a fundamental concept in organizational studies, with multiple definitions emphasizing its role in structuring and exercising power. According to Reberioux (9), governance refers to the organization and deployment of power within organizations. Similarly, Rajan and Zingales (10) define governance as the set of mechanisms for allocating and exercising power or hierarchical authority. These definitions underscore governance as both a structural and functional phenomenon, central to the stability and efficacy of organizational frameworks.

However, these authors, along with others, critique the traditional shareholder-centric view of governance (11). This perspective, which focuses exclusively on maximizing shareholder value, is increasingly seen as narrow and insufficient in addressing the complexities of modelrn organizations. Instead, scholars argue for a broader view of governance that emphasizes the distribution of power and the equitable allocation of value among all stakeholders. This broader approach frames governance as a mechanism for preventing conflicts and ensuring the alignment, or convergence, of utility functions among various actors within the organization (12).

Governance, therefore, is not merely about operational oversight but encompasses a set of rules and structures designed to address critical organizational concerns. These include the configuration of ownership, protection of minority interests and creditors, and the system of administration and control (13). This framework highlights the interplay between different levels of authority, including the executive management, the board of directors, and the shareholders. Such an understanding situates governance within a broader negotiating perspective, where power dynamics and stakeholder interactions shape organizational outcomes.

This broader perspective on governance has been enriched by stakeholder theory, which emphasizes the importance of recognizing and engaging all relevant parties that affect or are affected by the organization's activities (14, 15). Stakeholder theory challenges the traditional shareholder-dominant model by proposing that organizations have a responsibility to balance the interests of diverse stakeholders, including employees, customers, suppliers, creditors, and the broader community. Khurram and Pestre (16) further emphasize that governance mechanisms should foster inclusivity and accountability, ensuring that organizational power dynamics do not marginalize minority interests or create inequities in decision-making processes.

By incorporating these perspectives, governance emerges as a dynamic, multidimensional construct. It transcends the simplistic confines of hierarchical authority and shareholder dominance to encompass a network of relationships and mechanisms aimed at achieving organizational coherence, fairness, and sustainability. This expanded understanding of governance is particularly pertinent in contexts characterized by complex stakeholder environments, such as nonprofit organizations and sport governance, where diverse interests must be carefully balanced to ensure long-term success.

## The governance in nonprofit sports organisation

The governance of nonprofit organizations is directly influenced by how these entities, particularly sports federations, are directed and managed. Stakeholders play a pivotal role in ensuring effective governance through sound management and informed decision-making (4).

Sports federations, as the primary nonprofit sports organizations at the national level, have emerged as key players in the political, social, and economic spheres worldwide. Consequently, it is crucial to examine the various governance models applied to these federations, beginning with an understanding of their organizational structure.

Tunisian Sports federations operate within a liberal organizational framework due to their associative status. They are governed by a dual legal structure: externally, they adhere to the rules and regulations imposed by international federations, while internally, they maintain governance systems specific to each federation. However, state intervention often occurs in the name of the public interest, creating a dynamic relationship between the state and the federations. In such cases, the state defines and regulates sports policies, which means that sports federations are not entirely autonomous from an organizational standpoint.

Dual governance structure in Tunisian federations—combining internal self-regulation and external state oversight—creates both opportunities and challenges. On one hand, state involvement brings financial stability and alignment with national priorities. On the other, it can limit strategic autonomy, slow decision-making, and create tensions with international standards that demand independence. This hybrid model often places federations in a delicate position, balancing bureaucratic accountability with the need for operational flexibility.

According to Bayle (17), governance is a central issue in nonprofit organizations because their objectives are not driven by a single indicator, such as profit in corporate entities. This distinct nature requires the identification of key characteristics that differentiate national sports federations from traditional companies.

Several scholars, including Charreaux (18) and Chantelat (19), have highlighted the unique features of sports federations as hybrid organizations:

1. Nonprofit Purpose

The primary objective of sports federations is not the pursuit of profit but rather goals of a societal and extra-economic nature. These include promoting sports participation, fostering community engagement, and contributing to public well-being.

2. Blended Financial Resources

Sports federations rely on a mix of funding sources. These include direct and indirect public financing, such as government grants or subsidies, alongside revenues from commercial activities like sponsorships, licensing, and event organization. This hybrid financial model underscores their dual reliance on public and private sectors.

3. Dual Staffing Structure

The workforce in sports federations is characterized by a mix of paid professionals and volunteers. In some cases, staff members are seconded by the state, local authorities, or even public companies. This dual structure presents governance challenges related to managing diverse expectations, roles, and levels of commitment.

4. Affiliation with Supranational Regulatory Systems

Sports federations operate within a multi-layered governance framework. They adhere to regulations set by continental sports unions, international federations, and the International Olympic Committee (IOC). This membership allows them a degree of autonomy from national public authorities while also obliging them to conform to global standards and practices.

These characteristics illustrate the complexity and hybrid nature of national sports federations. Their governance systems must navigate the interplay between public accountability, financial sustainability, and adherence to both national and international regulations.

Moreover, the governance models of sports federations must balance the diverse interests of stakeholders, including governments, sponsors, volunteers, and athletes. Transparency, accountability, and inclusivity are essential to maintaining public trust and ensuring that these organizations achieve their societal missions.

By understanding the specificities of sports federations, we can develop governance frameworks that not only address their unique challenges but also enhance their ability to contribute positively to societal and global sports ecosystems.

## The organisational performance on sports organisations

In a sports organization, organizational performance relies on various categories of individuals responsible for designing and managing sports activities. These include volunteer leaders, technical managers (such as coaches and technical directors), and employees (such as secretaries, managers, or sports directors). According to Knauft et al. (20), the performance of an organization is influenced by three key operating principles of the Board of Directors: collective spirit, enthusiasm, and the dedication of its members.

Building on the idea that organizational performance is determined by multiple criteria, Pawlak and Flynn (21) highlighted that the performance of Boards of Directors is assessed based on their ability to manage funds and oversee political and social relationships. In other words, good governance is central to the evaluation of organizational performance.

Chappelet and Bayle (22) emphasized the importance of identifying governance models and improving governance practices in national sports federations. These practices play a critical role in their strategic and organizational operations, directly influencing their overall performance. It follows that the type or model of governance adopted by a national sports federation significantly impacts its effectiveness and efficiency.

## The governance models of sports organization

Mayaux (23), followed by Bayle (17), identified four distinct types of governance models within organizations. These are the “strong presidential” model, the “couple's presidential” model, the “exploded presidential” model, and the “managerial presidential” model. In the following sections, we will explore each model in detail.

1. The “Strong Presidential” Model

The strong presidential model is characterized by the president assuming a dominant and pivotal role in decision-making and the overall functioning of the organization. In this governance model, the president emerges as the central figure, concentrating and centralizing decision- making authority. As noted by Lawrence and Lorsch (24), integration within such organizations is achieved through the management of power, which involves specific actions aimed at facilitating the organization's operations during attribution conflicts or challenges stemming from human behavior.

This model relies on a tightly centralized structure where the president depends on one or two key individuals for internal control and strategic alignment. Typically, these individuals are the national technical director and the general secretary, with whom the president maintains close, daily communication. These key figures are instrumental in shaping and executing the organization's strategy. Key characteristics are summarized in Table 1.

Bayle (17) highlights that this model of governance places significant emphasis on the authority and direct involvement of the president, making it highly centralized and reliant on a select few for decision-making and organizational management.

This model is presented and simplified by the diagram below. This structure is illustrated in Figure 1:

This model of governance is closely associated with a “control” board of directors. According to Mayaux (23), a board of directors functioning as a “control device” takes on the primary responsibility for managing the sports organization and overseeing the work of its employees. In this setup, board members act as employers, exerting authority and playing a dominant role in organizational decision-making.

In some cases, this concentration of power may arise due to the disengagement of elected members or permanent staff from federal management activities. Such disengagement can result in a governance structure where the board assumes an even greater role in directing and controlling the organization's operations.

2. The “couple’s presidential” model

The “couple's presidential” model of governance is characterized by a strong presidential authority, complemented by the involvement of two or three key professional collaborators. These typically include the national technical director, who supports the president in technical matters, and the general secretary, who plays a crucial role in assisting with strategic decision- making and ensuring its implementation. In this model, decision-making power is distributed almost equally between the president, the general secretary, and the national technical director. This collaborative leadership structure ensures that the president does not act in isolation. The governance structure is detailed in Table 2 but rather in close coordination with these key figures, each of whom brings specific expertise to the decision-making process.

The balance of power helps streamline strategic decisions, particularly those that require both technical and administrative considerations.

See Figure 2 for a schematic of the couple's presidential model. This model of governance fosters a dynamic, cooperative approach where strategic initiatives are collectively developed and executed, with each member of the leadership team contributing to the broader organizational vision. It encourage more balanced distribution of responsibility, with the president still holding a dominant leadership position, but with support from trusted and skilled collaborators who share in the decision-making process.

This model of governance is closely associated with a board of directors functioning as a “supporting tool”. According to Mayaux (23), the board of directors in this model provides advice, guidance, and assistance, and is perceived by employees as both colleagues and facilitators. The board is seen as a forum for exchange, reflection, and proposal, serving as a support group for the organization's leadership.

In this governance structure, the arrival of a new president often requires the establishment of legitimacy, which is typically achieved through the support of a long-standing, influential figure—usually the national technical director or administrative director—who has been with the organization for several years. These individuals typically possess a deep understanding of the technical and administrative aspects of the federation. This information asymmetry, where the director has more knowledge and experience, plays a key role in shaping the president's leadership approach. As a result, the new president is often inclined to collaborate closely with this figure to solidify their position of power.

In this system, the balance of power between the two key figures—the president and the director—can shift over time, sometimes leading to the emergence of authoritarian presidential power. One of the risks inherent in such a governance structure is the potential for the president's personal objectives to overshadow the federation's broader goals. For instance, the desire for re-election by the General Assembly may become a dominant priority, resulting in irrational management of the association's affairs from a sports performance perspective. In some cases, to secure the support of a majority of members, the federal president and their team may organize an electoral campaign at the expense of sports policy, diverting focus from the federation's primary objectives.

Despite these risks, there is a necessary symbiosis between the president and the director in this system. The national technical director or administrative director relies on the democratic legitimacy of the president, while the president, in turn, depends on the technical expertise and insight provided by the permanent director. This mutual reliance fosters a natural alliance, built on a compromise between the political and technical dimensions of the federation's leadership.

3. The “Exploded Presidential” Model

The “exploded presidential” model is a governance structure in which the president deliberately surrounds themselves with several salaried directors, each responsible for the technical and strategic management of specific areas within the organization. In this setup, the president assumes the role of a coordinator, overseeing the entire organization while maintaining close management of a dedicated team of volunteer leaders. This division of labor leads to a specialization of tasks among the main volunteer leaders on the board, with each leader focusing on distinct responsibilities that align with the overall organizational strategy.

In this model, the president's leadership is more coordinative than direct, as the salaried directors handle specific sectors of the organization's operations. These directors bring technical expertise and strategic insight to their respective areas, while the president focuses on ensuring cohesion and alignment across the different sectors. This form of governance promotes a clear distribution of responsibilities. Operational divisions can be seen in Table 3, with a stronger emphasis on delegation and professional management at various levels of the organization.

While the president remains the central figure, this model of governance allows for greater specialization, potentially enhancing operational efficiency and allowing for more informed decision-making in each area of the federation's activities.

The president coordinates all the directors and puts himself in a position of general manager.

The exploded model is diagrammed in Figure 3. In the “exploded presidential” governance model, the president works closely with a board of directors that, according to Mayaux (23), acts as a “facade.” In this structure, the role of the board of directors is quite limited and formal, often reduced to endorsing decisions that have already been made by employees or salaried directors. Board meetings are typically procedural, where decisions that have been formulated behind the scenes are presented for formal approval.

The federal office in this governance model is composed of a team that is strongly aligned with the president, often working directly for and through the president. Key responsibilities for overseeing and managing specific departments or activities are entrusted to the principal officers within the office, typically the vice-president and treasurer, who are considered the president's trusted collaborators. These individuals may hold various critical functions, such as managing sensitive political issues or overseeing certain projects. In some cases, they may provide informal coordination for the entire structure, especially during the physical absence of the president.

This model of governance is more complex than the strong presidential or couple's presidential models because it requires a delicate balance of power among salaried directors, volunteer leaders, and between volunteers and employees. The president's role becomes central, particularly in arbitrating prerogatives and responsibilities across different sectors. The president's authority is often contingent on a high degree of trust and coordination. In cases where presidential legitimacy is contested, there is a significant risk of sclerosis, dysfunction, or inertia in the federation's operations, as internal power struggles can hamper the decision- making process and overall effectiveness.

This model highlights the importance of clear and stable leadership, as well as effective communication and collaboration among all members of the governance structure to prevent breakdowns in functioning.

4. The “managerial presidential” model

The “managerial presidential” model of governance is characterized by the centralization of decision-making power in the hands of a general manager (GM) or National Technical Director (NTD), rather than a volunteer leader such as the president. In this system, the decision-making authority is typically formalized or tacitly accepted by the board of directors, which plays a more passive, “facade” role. The board's functions are largely symbolic, with its members often approving decisions that have already been made by salaried staff, such as the GM or NTD.

In the managerial presidential model, the NTD or salaried director is vested with significant decision-making power. This individual may exercise both formal and informal control over the organization's direction and operations; decision roles and authority levels are shown in Table 4. Such directors often command a high degree of trust from key elected members, who implicitly accept the concentration of power in the hands of the salaried management. This is because the NTD or GM typically possesses the technical expertise and managerial experience necessary to guide the federation effectively, while the volunteer board members may lack the specialized knowledge required to make day-to-day decisions.

This model is commonly seen in organizations where there is a need for professional management and expertise in running the federation's operations. The board's role becomes largely one of oversight, but it is typically less engaged in the operational and strategic decision- making processes.

While this structure can bring efficiency and professionalism, it also creates a potential disconnect between the elected leaders and the salaried management. There is a risk that decision-making may become overly centralized, with the salaried director or manager wielding disproportionate influence, and elected members might feel excluded from critical decisions. This could lead to tension or a lack of alignment between the elected leadership and the professional staff, undermining the democratic legitimacy of the federation.

The managerial presidential model, therefore, requires careful balancing of power and a clear definition of roles to ensure that the elected leaders' authority is respected, while also allowing the professional managers the freedom to operate effectively. If not carefully managed, this model can lead to governance challenges where the influence of salaried staff eclipses the role of volunteer leadership.

This type of governance requires a high level of trust and delegation of significant responsibilities from the volunteer leaders, either willingly or reluctantly, and often without full awareness. The volunteer leaders place significant authority in the hands of an employee, or a person made available by the State, who, either formally or informally, holds the real decision- making power. This individual, typically the general manager or National Technical Director, effectively controls the organization's direction and operations, and their influence often extends beyond formal structures.

Refer to Figure 4 for the managerial presidential configuration. The director, as permanent dominant figure, can gain legitimacy through several factors, including their expertise, federal career (such as being a former champion), achievements (in sports or other areas), the acceptance of their authority by elected officials, and their seniority within the organization. These various foundations of power and legitimacy allow the director to maintain their position and status. The legitimacy of this dominant figure is often grounded in multiple and powerful sources, all working together to reinforce the significance of their role and authority.

This governance model is complex, as it involves both formal and informal prerogatives that must be carefully negotiated between the key players. The dynamics between these actors determine the flow of power and decision-making within the organization. However, this model can also be fragile, as it is heavily dependent on the position of the volunteer leaders and their relationship with the dominant actor. A shift in leadership or the departure of the permanent dominant figure can pose significant risks to the stability of the governance system.

## Research methodology

Data collection is conducted in two stages. The first stage involves a study and exploratory interviews to identify the federations and their specific characteristics. This phase also helps to determine the categories of individuals to interview, as well as to define the parent population and the sample.

The original set of questions and performance measures were designed by Geeraert (25) and further developed in the National Sports Governance Observer 2 report. The NSGO indicators have been peer-reviewed and are widely recognized for their contribution to sports governance research.

From a pool of national sports federations within the same country, 20 federations were selected for the study. These federations are all part of the Olympic summer program, ensuring that the sample focuses on those with a significant emphasis on sports performance and financial resources. The decision to focus on a single country, Tunisia, was made to control for historical, economic, and cultural differences that could affect the findings. By limiting the study to these 20 Olympic federations in Tunisia, the research aims to provide a more focused and coherent analysis of governance in the context of high-performance sports.

In the second step, the final data collection is carried out involving both volunteers and paid staff from the 20 selected sports federations. The parent population consists of the individuals directly involved in the governance of the federations, which includes both volunteers and paid staff members. The survey targets the president, members of the executive committee, and key employees (such as the General Secretary and National Technical Director) of each federation.

An online survey is used for data collection due to its advantages, including the speed of dissemination, the ability to directly collect responses that are easily analyzable through data analysis software, and the savings in both time and cost. The survey is accompanied by an explanatory note outlining the subject of the study and an invitation to participate.

The final questionnaire is introduced in SPSS to analyze data, which is employed for both data collection and analysis. To identify the profiles of the respondents, univariate statistical analyses are conducted, with flat sorting applied to each variable. The results of reliability tests (using Cronbach's alpha) are utilized to assess the uni-dimensionality or multi-dimensionality of the variables.

These tests help to refine the conceptual model, allowing for the inclusion of relevant dimensions for each explanatory variable and the variable to be explained.

## Findings

Each model of governance in sports federations was tested for its impact on organizational performance in order to determine whether the governance model adopted either hinders or, conversely, positively or negatively influences the federation's performance. The statistical results reveal a significant correlation and a positive impact of each model of governance on organizational performance. To obtain these results, a questionnaire was sent to 80 respondents from various roles within different federations. The sample consisted of 25 General Secretaries (20 men and 5 women), 25 Presidents (23 men and 2 women), and 30 National Technical Directors (28 men and 2 women). The operationalization of the constructs was clearly defined, and the scales of measurement for both the explanatory variables and the dependent variable were carefully specified.

To test the hypotheses, an exploratory factor analysis was performed to examine the dimensionality of the variables. The results show that in 33% of federations, strategic decisions and managerial operations are made through coordination between the various actors involved. Additionally, 24% of federations operate under a “couple” or “exploded” presidential model of governance. In the remaining federations, the president is the sole decision-maker. These findings are in line with the study conducted by Bayle (17), which emphasizes the diverse governance structures within federations.

Despite these positive influences, the results also highlight a paradox: no single model of governance emerges as predominant. All four models of governance positively influence organizational performance, but the extent of this influence seems to be closely tied to the political evolution of each country, as suggested by Bayle (17).

Furthermore, the data suggests that the presence of women on the board does not significantly affect the organizational performance of the sports federations. The survey revealed a significant gender imbalance: only 10 of the 80 respondents were women. While statistical tests suggest that gender does not significantly affect organizational performance, this finding should be interpreted with caution. The small sample size of female participants likely reflects deeper structural barriers to women's leadership in sport governance, such as unequal access to positions of power and entrenched gender norms within federations.

This observation challenges some commonly held assumptions about gender diversity as a key factor in improving performance in governance structures.

## Discussion

This study highlights the significant impact of governance models, as conceptualized by Bayle (17), on the organizational performance of nonprofit organizations, particularly national sports federations. It specifically examines how these governance structures influence both sports results (26) and the financial health of federations. By linking governance practices directly with performance outcomes, the research emphasizes the critical role of leadership structures in determining the success of sports organizations.

In the context of Tunisian sports federations, the government's influence presents both positive and negative effects on governance. A comprehensive exploration of these trade-offs provides valuable insights into the balance between accountability and autonomy.

State funding often represents the primary financial resource for sports federations, particularly in countries where the private sector plays a limited role in sports financing. This funding ensures the sustainability of operations and facilitates the implementation of development programs. Government involvement may reinforce transparency and ethical standards by imposing strict financial and operational controls. Regular audits and reporting requirements help reduce the risk of corruption and mismanagement. Moreover, government oversight aligns the objectives of sports federations with national sports policies and public interest goals, such as youth development, gender equality, and social inclusion.

However, government control can also restrict the decision-making capacity of sports federations, particularly regarding leadership appointments, strategic priorities, and financial allocations. This may hinder the long-term vision of federations. Political interference may compromise the independence and integrity of federations, especially if leadership appointments are based on political affiliations rather than merit. Additionally, bureaucratic processes can delay decision-making, reducing the agility of federations to respond to emerging challenges or opportunities.

Over the past decade, Tunisia has undergone substantial political, legislative, and social changes, which have significantly influenced the governance of sports organizations (16). One key development in this transformation has been the revision of statutory frameworks governing sports organizations, influenced by decrees issued by the Tunisian National Olympic Committee and the Ministry of Youth and Sports. These decrees mandated the re-election of all members of sports organizations, with one-third of executive bureau members appointed by the Ministry of Sport, while the remaining two-thirds are elected.

This reform aims to improve governance by reinforcing the principle of independence within sports structures.

The governance reforms have yielded tangible improvements in performance, first observed during the 2012 London Olympic Games, where the Tunisian Swimming and Athletics Federations won two gold medals. In 2016, the Tunisian Taekwondo, Fencing, and Wrestling Federations secured three Olympic medals at the Rio de Janeiro Games. Furthermore, the Tunisian Judo Federation was awarded the Golden Torch of Governance in 2018 for exemplary governance practices, following similar recognitions granted to other national federations by the National Olympic Committee of Tunisia (27). While these achievements coincide with governance reforms, it is important to acknowledge that Olympic success is a multi-causal outcome. Athletic performance at the elite level depends on a range of factors beyond governance, including talent identification, coaching quality, access to facilities, investment in athlete development, and long-term planning. Thus, while improved governance may have facilitated organizational stability and strategic direction, it should be viewed as one contributing factor among many. Overstating this connection risks ignoring the complex ecosystem that underpins elite sports success.

These achievements underscore the positive impact of governance reforms on sports performance. While Tunisia has made notable strides in governance reforms, similar statutory models are observed in developed countries like Belgium and Canada, where nonprofit sports organizations remain largely under the supervision of the state. In these countries, the full board of directors is often appointed by the Ministry of Sports or the National Olympic Committee. This model is also prevalent in several Gulf countries, including Kuwait and Bahrain. The International Olympic Committee (IOC) has called for a clear separation between politics and sports, advocating the adoption of good governance principles such as transparency, efficiency, and effectiveness (28). The IOC also promotes the inclusion of qualified members on boards of sports federations. Tunisia, in line with these recommendations, has sought to implement such practices to improve governance quality and organizational performance.

The state plays a pivotal role in the governance of sports federations, defining and regulating sports policies while contributing significantly through financial support (subsidies), human resources (civil servants), and infrastructure (sports facilities). Consequently, sports federations are not entirely free to devise their own strategies for growth and development.

The organizational performance of sports federations is often gauged through a framework of inputs, throughputs, and outputs. Inputs typically refer to funding, throughputs involve resource management and allocation processes, and outputs reflect outcomes such as international sports achievements. Given the state's substantial role, performance metrics often emphasize financial health (throughputs) and sports success (outputs), particularly in terms of medals at major international competitions.

This state-federation relationship creates tension between short-term and long-term goals. On the one hand, the state expects federations to demonstrate annual success through measurable outcomes. On the other hand, developing high-level athletes requires significant investment of time and resources, challenging the long-term planning necessary to build sustained success in global competitions. This dual pressure complicates the governance landscape, as federations navigate the shifting priorities set by the state.

In summary, while Tunisia has made progress in improving the governance of its sports federations, external pressures from the state and international bodies like the IOC continue to shape governance structures and their impact on performance. The ongoing balance between meeting short-term goals and investing in long-term success remains a key challenge for Tunisian sports governance.

A comparative discussion between different governance regimes provides useful insights. In **France**, most federations operate under democratic structures with moderate state influence, though funding still comes largely from public sources. **Norway** offers an interesting contrast, with federations enjoying both generous public funding and strong organizational autonomy. These models demonstrate that autonomy and public support are not necessarily incompatible.

In contrast, **Morocco** and **Algeria** show a more centralized approach, where government authorities have direct influence over leadership appointments and strategic decisions.

In the **Gulf countries** (e.g., **Kuwait**, **Bahrain**), the governance structures are heavily dependent on state direction, often blending political and administrative control within federations. These cases reveal how governance models are shaped by broader political systems, and how different configurations of autonomy and control affect organizational outcomes. A deeper comparative lens could guide reform strategies adapted to national contexts.

This comparative analysis highlights the diverse governance models in sports federations and their implications for organizational performance, providing a broader context for understanding the Tunisian experience.

## Conclusion

Tunisian sports organizations pursue a range of non-financial objectives related to their sporting mission, which necessitates a distinct approach to performance management compared to profit-oriented companies. Additionally, their governance often relies, in part or entirely, on volunteers, making it essential to adapt performance management strategies accordingly. The study of governance models in nonprofit organizations, particularly sports associations, represents a promising avenue for research. Governance is a crucial challenge for sports federations aiming to enhance their operations and management practices.

This study holds significant value for improving the strategic and organizational functioning of private organizations entrusted with a public service mandate. By examining the managerial practices within sports federations, the research aims to contribute to the improvement of sports management and to expand scholarly work in this area, particularly given the limited research conducted in Tunisia. This study sheds light on the governance models employed by national sports federations and underscores the necessity for structures capable of succeeding in this competitive field. Furthermore, it demonstrates how the governance models, as conceptualized by Bayle (17), influence organizational performance, as evidenced by both sports achievements and financial outcomes within these federations. The data analysis highlights a positive impact of each governance model on organizational performance, regardless of the specific governance approach adopted.

However, each governance model leads to different internal dynamics. For instance, the managerial presidential model emphasizes operational efficiency by delegating power to salaried directors, but may reduce the involvement of elected officials. The couple's presidential model supports strategic co-leadership, fostering shared expertise, while the exploded presidential model distributes responsibilities across multiple actors, allowing for specialization but requiring high coordination. Finally, the strong presidential model concentrates authority, which can ensure quick decisions but may reduce transparency and collaboration. These distinctions illustrate how governance structures influence daily processes, leadership dynamics, and organizational culture

Like any research, this study has its limitations. Methodologically, the study draws primarily on the governance model developed by Bayle (17), though other models could have been employed, such as those proposed by Ferkins, Shilbury, and McDonald (29) or Winand et al. (8). While Bayle's model provides valuable insights, it has certain limitations, particularly in its heuristic value. It is more descriptive and normative than explanatory, and this aspect makes it somewhat weak in delineating the precise boundaries of the four governance models it proposes. These limitations could be addressed in future research by incorporating additional models or refining the existing framework to enhance its explanatory power.

This study contributes to the promotion and development of the interrelationships between key actors in decision-making processes within sports federations. These interactions have a direct impact on the overall functioning and efficiency of these organizations. In particular, the study emphasizes the critical relationship between national sports federations and the state, a dominant stakeholder in sports governance. The findings highlight how state involvement affects the autonomy of federations and influences their decision-making processes. This relationship underscores the need for federations to balance external pressures with the internal governance dynamics that shape their strategic direction.

The conclusions drawn from this study and the results obtained pave the way for future research exploring new dimensions of governance in nonprofit sports organizations. Future studies could focus on comparative investigations of different governance models across various countries or regions, examining how governance influences not just performance outcomes but also organizational sustainability and long-term development. Additionally, a deeper understanding of the characteristics of governance—such as the role of volunteer leaders vs. paid staff, the influence of external stakeholders, and the impact of legislative frameworks— could provide a more nuanced perspective on how these organizations can optimize their governance structures to achieve both short-term and long-term goals. While Bayle's typology has proven useful for describing governance configurations, it remains primarily descriptive. Future research could benefit from incorporating alternative or complementary frameworks, such as Winand et al. (8), which emphasize organizational performance dimensions, offer a unified model linking performance outcomes in nonprofit sport organizations, providing a more integrative understanding of how governance affects results, or Ferkins and Shilbury's strategic governance model, which focuses on board engagement in long-term planning and stakeholder management. A comparative or mixed-model approach would allow for a more nuanced understanding of how governance mechanisms function across contexts and over time.

Ultimately, this study serves as a foundation for future research in sports governance, providing valuable insights into the relationship between governance models and organizational performance. Board involvement in long-term strategic planning is emphasized in governance literature (28), highlighting the need for proactive leadership and stakeholder engagement in sport federations. The results emphasize the importance of strategic leadership and the governance framework in achieving the goals of sports federations, while also acknowledging the ongoing challenges posed by the complex interactions between various stakeholders. The development of more effective governance practices will be crucial for the continued success and evolution of sports federations, particularly in nonprofit settings.

## Data Availability

The original contributions presented in the study are included in the article/Supplementary Material, further inquiries can be directed to the corresponding author.
